# Predictors of Problematic Smartphone Use: An Examination of the Integrative Pathways Model and the Role of Age, Gender, Impulsiveness, Excessive Reassurance Seeking, Extraversion, and Depression

**DOI:** 10.3390/bs8080074

**Published:** 2018-08-14

**Authors:** Lewis Mitchell, Zaheer Hussain

**Affiliations:** College of Life and Natural Sciences, School of Human Sciences, University of Derby, Derby DE22 1GB, UK; lewis-mitchell@live.co.uk

**Keywords:** problematic smartphone use, impulsiveness, excessive reassurance seeking, depression, extraversion

## Abstract

*Background*: The progression of mobile phone technology has led to the development of multi-functional smartphones providing access to features such as social media, e-mail, and videos alongside the basic functions of a mobile phone. Increasing amounts of research has explored the potential addictive nature of smartphones to develop a theoretical framework that describes personality factors related to problematic use. The present study examined the Integrative Pathways Model and the effect of age, gender, impulsiveness, excessive reassurance seeking, extraversion, and depression on problematic smartphone use. *Method*: A total of 147 smartphone users (mean age = 30.96, SD = 12.97, 69.4% female) completed an online survey comprising of measures of problematic smartphone use, excessive reassurance seeking, extraversion, depression, and impulsiveness. *Results*: Age, impulsiveness, excessive reassurance seeking, and depression were all significantly related to problematic smartphone use, however extraversion was not significantly related. Furthermore, age and impulsiveness were significant independent predictors of problematic smartphone use. No gender differences were found. *Conclusions*: The findings presented several factors that predict problematic smartphone use, implications and suggestions for future research are discussed.

## 1. Introduction

The introduction of smartphones has provided users with a more comprehensive mobile device; providing access to social networking sites, the internet, and numerous applications whilst maintaining the basic functions of a mobile phone. The positive impact of this technological change has seen smartphones help to increase dialogue between politicians and citizens [1], improved business practices [2], and enhanced healthcare services [3,4,5]. Accessibility to smartphones has also greatly increased in recent years with 4.23 billion smartphones being used worldwide, that figure being expected to surpass 5 billion by 2019 [6]. Simultaneously to this increase in smartphone use, researchers have been studying the negative consequences of problematic smartphone use (PSU). These negative consequences include symptoms of depression and anxiety [7,8], detrimental effects on attention [9], financial problems [10], sleep disturbances [11], dangerous use [12], and problematic social and academic behaviours [9,13]. The increase in research exploring PSU in the past decade has also brought about numerous different conceptualisations of smartphone use; including mobile phone addiction [14], problematic mobile phone/smartphone use [15,16], mobile phone dependence [17], and nomophobia [18,19]. This has stimulated discussion as to whether PSU can be considered as a potentially addictive behaviour.

Dependence to smartphones has been classified as a technological addiction [20,21], which involves a dependence on a non-substance and human-machine interaction. Technological addictions fall under the broader category of behavioural addictions [22]. Behavioural addictions are a contentious topic (internet gaming disorder is the only recent non-substance-related disorder proposed for inclusion in the fifth edition of the Diagnostic and Statistical Manual of Mental Disorders DSM-5; [23]), with many critical questions remaining unanswered [24]. Research to establish the addictive properties of various behavioural addictions is warranted. Recent research [25] has established that smartphone addiction has several similar characteristics to DSM-5 substance-related disorder (i.e., compulsive behaviour, functional impairment, withdrawal, and tolerance). Research exploring behavioural addictions has led to the identification of several core components of addiction, these include; salience, mood modification, tolerance, withdrawal symptoms, interpersonal and intrapersonal conflict, and relapse [21,26,27]. Researchers have used these core components to examine addiction to smartphones [28,29]. Critically, The World Health Organization [30] considered excessive smartphone use as a public health concern which requires further investigation. To date, few studies have examined PSU. Existing studies have been conducted in South Asian countries [25,31,32]. Little is known about the potential predictors of problematic smartphone use.

A theoretical framework is essential to facilitate development in establishing the aetiology and manifestations of PSU. Research [33] has suggested that overuse of short message service (SMS) can result from an extraversion pathway; overuse is dictated by a desire to socialise and communicate with others and a neurotic pathway; driven by anxiety about relationship maintenance. In parallel with this and following an increase in research investigating PMPU, the integrative pathways model ([15]; IPM) proposes three pathways that lead individuals to engage in PMPU. The IPM [15] also differentiates between addictive, anti-social, and risky patterns of mobile phone use. This is one of the most recent proposals for a theoretical framework for PMPU and can be applied to the more modern behaviour of PSU. The IPM is a comprehensive framework, based on empirical data that supports the pathways, which aims to stimulate research into PSU. The model can support and guide research in the field of smartphone use. 

The first pathway of the IPM [15] is excessive reassurance seeking, this relates to individuals who engage in PSU to obtain reassurance from others and maintain relationships. Excessive reassurance seeking was first described in the interpersonal theory of depression [34]. It has subsequently been defined as “the relatively stable tendency to excessively and persistently seek assurances from others that one is lovable and worthy, regardless of whether such assurance has already been provided” [35] (p. 270). Previous research infers that concerns about relationship maintenance is related to excessive and uncontrolled SMS use [33,36], exemplifying how communication using mobile phones can act as a medium for reassurance. Further to this, it is hypothesised that general and social anxiety are also both associated with PSU and excessive SMS use [8,10,37,38]. This suggests that increased anxiety–alongside poor self-esteem [39], insecure attachments [40], and neuroticism [41]—all contribute to a greater need for reassurance, which in turn leads to an addictive pattern of PSU. Despite this, it is important to consider that the aforementioned research is based on established risk factors around the excessive reassurance pathway of the IPM [15], not excessive reassurance seeking specifically. 

The second pathway of the IPM [15] is impulsiveness. This corresponds to individuals in which PSU is the result of poor impulse control, leading to the uncontrolled urge to use their smartphone. Characterised by urgency, a lack of planning, lack of perseverance, and sensation seeking [42], impulsiveness can lead to antisocial use including cyberbullying [43], the use of mobile phones in banned/socially unacceptable areas [10]; addictive use exemplified by addictive patterns of smartphone use being associated with high-impulsivity traits such as low self-control, lack of premeditation and symptoms of attention deficit hyperactivity disorder (ADHD) [44,45,46], and also risky use such as dangerous driving or “sexting” (exchange of sexually suggestive images/messages) [10,47]. However, the diversity of definitions and measures used in previous research has led to ambiguity surrounding the individual facets of impulsiveness and their subsequent relationships with PMPU and PSU. 

The third pathway of the IPM [15] is extraversion. This pathway corresponds to individuals who display symptoms of dependence to their smartphone and whose over-usage is driven by a consistent desire to build and maintain relationships, communicate with others, and a constant need for stimulation and reward. An abundance of research has inferred an association between extraversion and PMPU/PSU [33,41,48]. It is suggested that the extraversion pathway, similar to impulsiveness, can lead to addictive, antisocial, and risky patterns of use. Notably, the sensation seeking traits that are typical of extraverted individuals [49] can lead to antisocial and risky patterns of behaviour such as cyberbullying [43] and sexting [50]. Despite this, some recent studies have found either a negative or no relationship between extraversion and PMPU [51,52]. Further research is needed to establish extraversion as a predictor of PSU. Furthermore, it has been acknowledged that research is still in its early stages and that the IPM should serve as a medium for development of smartphone research rather than a complete theory [15].

Whilst excessive reassurance seeking, impulsiveness, and extraversion all have strong evidence linking them to PMPU and PSU, the relationship of age and gender is more ambiguous. In age and gender there is evidence both inferring a link and contradicting one. For example, some evidence suggests that young individuals are more likely to exhibit PSU [41,48,53], whereas other research suggests no relationship between age and PSU [54]. Assuming that age is a predictor of PSU, it has been reported that younger individuals may be more inclined to engage in PSU due to poorer impulse control [55]. However, more research studies that include older populations are needed to clarify this. In terms of gender differences in PMPU, research suggests that women engage in the texting features of mobile phones significantly more than men [10], a similar result has been found among social media users [56]. Despite these findings, research has also found no evidence of gender differences [57]. Ultimately, more research is needed to establish whether age and gender are genuine predictors of PSU.

PSU has also been found to be comorbid with depressive symptoms [58]. Depression severity has been consistently linked to PSU [7,11,59], this is often attributed to individuals engaging in smartphone use as an escape mechanism to deal with depressive symptoms and negative emotions [60]. It has also been argued that PSU could have an indirect effect on depressive symptoms through lack of sleep and work-related symptoms [61,62]. The work/family border theory [63], as well as research [64], describes how a lack of boundaries between work and home life—enhanced by the multi-functionality of smartphones allowing work to be accessed at home—can lead to increased stress levels and, in turn, depressive symptoms. It has also been suggested that the relationship between PSU and depressive symptoms is bi-directional [65], whereby increased PSU is driven by depressive symptoms, and that increase in PSU leads to a concurrent rise in depressive symptoms—inferring a paradoxical relationship. Further reinforcing the link between PSU and depression, excessive reassurance seeking has been identified as a feature of depression [35,66] as described in the interpersonal theory of depression [34]—this relates back to the IPM [15]. 

Previous research has inferred relationships between PSU and the personality variables highlighted by the IPM [15], however there has been little evidence showing the extent of influence the different pathways have on PSU. It is important to test and develop proposed models to achieve an accurate theoretical base. Further to this, no existing research has directly investigated the three pathways outlined in the IPM, although numerous studies have explored factors around the three pathways. More research needs to consider the multifaceted nature of the personality variables being examined within the IPM. Given the current state of research in the area, the present study aims to examine the IPM [15] by exploring the degree of influence that each pathway, along with age, depression, and gender has on the likelihood of an individual displaying PSU. It is hypothesised that high impulsiveness, high excessive reassurance seeking, high extraversion, being of younger age, and high depression will all significantly predict PSU, and that there will be a significant difference between the levels of PSU in males and females. Considering the diverse negative consequences of PSU, it is important to further investigate its associated negative consequences to broaden understanding of the area and begin to formulate necessary interventions to prevent PSU. 

## 2. Method

### 2.1. Design

The present study adopted a cross-sectional correlational design exploring the relationship between smartphone use and personality characteristics. An online survey method was used in the present study. The variables under investigation included: (i) problematic smartphone use, (ii) impulsiveness, (iii) excessive reassurance seeking, (iv) extraversion, and (v) depression. Further to this, information regarding participant demographics (e.g., age, gender, ethnicity) and general smartphone use was collected.

### 2.2. Participants

A total of 153 smartphone users provided fully completed survey responses, 6 responses were removed after data cleaning (see analytic strategy below) and for violating recommended statistical parameters for sample sizes that are more than 100 [67,68]. This left a final sample size of 147 smartphone users (mean age = 30.96, SD = 12.97). The participants age ranged from 18 to 68 years, with 42 males (28.6%) and 102 females (69.4%), three participants did not disclose their gender. The ethnicity of the sample included White (85.7%), Black (1.4%), Asian (1.4%), mixed-ethnicity (4.8%), and other (4.8%), three participants did not disclose their ethnicity. 

### 2.3. Materials

Online survey software called *Qualtrics* was used to create the survey. The online survey consisted of five measures, these are described below; 

*The Modified Internet Gaming Disorder Scale Short-Form*

The Modified Internet Gaming Disorder Scale Short-Form (MIGDS-SF; [69]) was adapted to measure PSU. The scale consists of nine items adapted from the *Diagnostic and Statistical Manual of Mental Disorders* [23] criteria for Internet Gaming Disorder. Example items include ‘Do you feel pre-occupied with your smartphone behaviour?’ and ‘Do you systematically fail when trying to control or cease your smartphone activity?’ Items were rated on a 5-point likert scale (1 = never–5 = very often), with a sum score ranging from 9–45 [69]. Previous research using MIGDS-SF have reported very good internal consistency [28,29]. Internal reliability of the scale in the present study was very good (Cronbach’s α = 0.86). 

*The Depressive Interpersonal Relationships Inventory–Reassurance Seeking subscale*

The Depressive Interpersonal Relationships Inventory–Reassurance Seeking subscale (DIPI-RS; [70]) was used to measure excessive reassurance seeking. The DIPI-RS is a 4-item subscale extracted from the 24-item Depressive Interpersonal Relationships Inventory [70]. Example items include ‘Do you find yourself often asking the people you feel close to how they truly feel about you?’ and ‘Do you frequently seek reassurance from the people you feel close to as to whether they really care about you?’. Items were rated on a 7-point likert scale (1 = Never–7 = Always), responses were averaged across the four items for an overall score between 1 and 7, with a higher score indicating higher levels of excessive reassurance seeking. The internal reliability of the *DIPI-RS* was excellent (Cronbachs α = 0.92).

*Mini Markers Extraversion Subscale*

Participants levels of extraversion were measured using the Mini Markers Extraversion Subscale (MMES; [71]). The MMES, extracted from the 40-item Mini Markers personality scale [71], consists of eight descriptive, single-word items, of which four are reverse-coded. Participants use a 9-point likert scale (1 = extremely inaccurate–9 = extremely accurate) to describe how certain personality traits apply to them, example items including ‘Talkative’ and ‘Bashful’. A total score is acquired by reverse-coding necessary items and summing the scores, with a greater score indicating higher levels of extraversion. The internal reliability of the scale was very good (Cronbachs α = 0.86). 

*The Barratt Impulsiveness Scale*

The Barratt Impulsiveness Scale (BIS; [72]) was used to measure impulsiveness. The BIS is a 30-item scale featuring 11 reverse-coded items, scored using a 4-point likert scale (1 = Rarely/Never–4 = Almost Always/Always). Example items include ‘I plan tasks carefully’ and ‘I have racing thoughts’. A sum score is yielded ranging from 30–120, where a greater score indicates higher levels of impulsivity. The internal reliability of the *BIS* scale was very good (Cronbachs α = 0.80).

*The Beck Depression Inventory*

The Beck Depression Inventory (BDI; [73]) was used to measure depression levels. The scale consists of 21 items which are scored on a 4-point likert scale (0 = Not at all–3 = All the time). Example items include ‘I do not feel sad’, ‘I am sad all the time and I can’t snap out of it’, ‘I feel I may be punished’. Responses are totalled and produce a score between 0–63, whereby a score greater than 28 indicates severe depression. The internal reliability of the *BDI* in the present study was very good (Cronbachs α = 0.85).

*Smartphone User Behaviour*

To gain an understanding of participants smartphone use, participants were asked to state how long (in minutes) they spent on their smartphones in one session and to state their most frequently used smartphone feature. 

### 2.4. Procedure

A web link to the survey and a message outlining the purpose of the study was posted on numerous social networking sites (e.g., *Twitter*, *Facebook*) and online discussion forums (e.g., *Reddit.com*). Participants were also recruited using the university online Research Participation Scheme. After clicking on the link individuals were presented with a participant information sheet, consent form, and the online survey. The survey was presented in the same order for all participants; demographic questions, *MIGS-SS*, *BIS*, *MM*, *DIPI-RS*, *BDI*. Finally, a debrief sheet was presented which restated the purpose of the study, confidentiality of data, and right to withdraw. Data was stored in *Qualtrics* and then transferred to IBM SPSS 22 for analysis. 

### 2.5. Ethics

The study was carried out in accordance with the Declaration of Helsinki and adhering to the British Psychological Society ethical guidelines. The university’s ethics committee approved the study. All participants were informed about the study and all provided informed consent. 

### 2.6. Analytic Strategy

The analytic strategy involved cleaning the data set by inspecting cases that were missing or were partial responses, such cases were removed from the data set, data was screened by checking skewness, kurtosis, *z*-scores (no absolute *z*-values were above/below 3.29 [67]). After cleaning the data, a sample of 147 was achieved. Statistical analyses included (i) descriptive analysis of the main sample’s characteristics, (ii) gender differences in PSU were investigated using an independent samples *t*-test, (iii) average length of smartphone session was analysed using a Mann-Whitney *U* test, (iv) a bias-corrected and accelerated bootstrapped correlational design was used to compare the predictor variables (age, impulsiveness, excessive reassurance, extraversion, depression, and length of smartphone session) against the outcome variable (problematic smartphone use), and (v) a multiple regression analysis was then conducted using the above variables as predictor and outcome variables.

## 3. Results

### 3.1. Descriptive Statistics

Table 1 shows the descriptive statistical results for the study sample. The main findings were that the observed levels of PSU were low (mean = 17.14, SD = 5.69), levels of impulsiveness were moderate (mean = 61.3, SD = 9.38), levels of extraversion were moderate (mean = 43.91, SD = 11.84).

### 3.2. Smartphone User Behaviour

The mean length that participants spent on their smartphones in one session was 15.12 min (SD = 12.02). Participants reported that the most frequently used feature of their smartphones was Messaging (39.5%, *N* = 58), followed closely by Social Networking (38.1%, *N* = 56). Table 2 shows the most frequently used smartphone features among the study participants. An independent samples *t*-test was conducted to explore gender differences in PSU scores (*t* = −0.322, *df* = 60.509, *p* = 0.748, two tailed) and a Mann-Whitney *U* test to compare average smartphone session length (Mann-Whitney *U* (*n*_1_ = 41; *n*_2_ = 101) = 1864, *z* = −0.940, *p* = 0.347, two tailed) however both inferred no significant gender differences which did not support the study hypothesis. 

### 3.3. Correlational Analysis

Correlational analyses were performed incorporating the main study variables in the study to provide statistical insight prior to the main regression analysis. The analyses revealed that PSU was negatively related to age and positively related to impulsiveness, excessive reassurance seeking, depression, and average session length. The average length of smartphone session was also negatively related to age, and positively related to excessive reassurance seeking and depression. Table 3 shows the correlations between all the study variables. 

### 3.4. Predictors of Problematic Smartphone Use

To examine the associations between age, impulsiveness, extraversion, excessive reassurance seeking, and depression on PSU, a multiple regression analysis was conducted using the Enter method. Collinearity checks were performed using tolerance statistics (all greater than 0.2) and variance inflation factor (all less than 10, mean = 1.34) indicating collinearity was not a cause for concern. A Durbin-Watson test was also conducted to check adjacent residuals were not correlated (Durbin-Watson score = 2.11). 

The model produced a large effect size (*R*^2^ = 0.25, *R*^2^*_Adj_* = 0.23, *F* (5, 141) = 9.46, *p* < 0.001); indicating that the predictor variables accounted for a significant amount of the variance in PSU. The analysis showed that age (*t* (147) = −3.30, *p* = 0.001) was a significant negative predictor of PSU, impulsiveness (*t* (147) = 3.02, *p* = 0.003) was a significant positive predictor of PSU. However, extraversion (*t* (147) = −0.95, *p* = 0.367), excessive reassurance seeking (*t* (147) = 1.82, *p* = 0.057), and depression (*t* (147) = 0.53, *p* = 0.617) were not significant predictors of PSU (Table 4). The results partly support the study hypotheses, indicating that younger individuals and those who score high on impulsiveness are more likely to exhibit problematic smartphone use.

## 4. Discussion

The present study explored the associations between problematic smartphone use and impulsiveness, excessive reassurance seeking, extraversion, depression, and age. The results revealed that age and impulsiveness were both significant predictors of PSU. More specifically, the findings revealed that age was a significant negative predictor of PSU, whereby younger individuals are more likely to display symptoms of problematic smartphone use. This corresponds with previous research findings that have reported an inverse relationship between age and problematic use [41,48,53]. The findings showed that age was negatively associated with average smartphone session length, suggesting that younger individuals are typically spending longer periods of time using their smartphones in one session, which may in turn increase the likelihood of problematic use. This notion is supported by previous research inferring that increased time spent on smartphones is associated with PSU [28,74]. No significant differences were found between gender, PSU levels, and smartphone session length. This adds to the already equivocal body of research exploring the effect of gender on mobile phone/smartphone behaviour [10,57]. Future research should consider exploring gender differences in relation to the numerous functions of smartphones, such as messaging and gaming, as these could account for the inconsistency in research investigating gender differences in overall smartphone behaviour.

In terms of the IPM [15], the study findings provide support for its application. The findings suggest that an impulsive pathway can lead to patterns of problematic use, as there was a positive association between these two variables, further establishing high impulsiveness as a principal characteristic of PSU. This is in line with previous research which found that individuals who are more likely to act impulsively will typically engage in higher levels of PSU [10]. This could be the result of high-impulsiveness individuals frequently failing to control urges to use their smartphones. Together, the findings of the study in relation to age and impulsiveness provide strong support for the notion that younger individuals are more likely to exhibit PSU due to their poorer impulse control [55]. Despite this, there was no association found between impulsiveness and average smartphone session length. This may be due to the lack of perseverance that is associated with impulsiveness [42], meaning that individuals cannot remain focused on a task, such as using their smartphone, for extended periods of time. Future research could consider the individual facets of impulsiveness alongside other potential predictors such as obsessive-compulsive disorder and their respective associations with PSU.

Bivariate correlations showed that higher levels of excessive reassurance seeking was positively associated with increased PSU suggesting that as an individual’s need for reassurance increases, their problematic use of a smartphone also increases. These findings indicate that the excessive reassurance pathway outlined in the IPM [15] is plausible, despite falling marginally short of being a significant predictor in the regression analysis. Whilst previous research has tended to focus on risk factors of excessive reassurance seeking—including anxiety [8,38] and neuroticism [41]—the present study is the first to provide evidence for an association between PSU and excessive reassurance seeking as its own trait. Excessive reassurance seeking was also found to be positively related to average smartphone session length suggesting that individuals who frequently seek reassurance are spending extended periods of time using their smartphones. This could be indicative of the addictive pattern of use that the excessive reassurance pathway is proposed to manifest as, with individuals spending longer periods of time using their smartphones to seek reassurance in order to satisfy the tolerance phenomenon—although research with problematic users is needed to further explore this notion.

Interestingly, and contradicting the IPM [15], the study found that extraversion was not a significant predictor of PSU, contradicting the majority of previous research which infers an association between extraversion and problematic use [33,41,48]. These findings could be attributed to the multi-functionality of modern smartphones. Whilst older devices were limited to more social functions such as text messaging and phone calls, contemporary smartphones also include functions such as gaming and internet use which introverted individuals may feel more inclined to use. Arguably, this is reflected in the fact that more recent research has found either inverse or no associations between extraversion and problematic use [51,52]. This provides a case for research to focus more exclusively on certain types of smartphone usage. The IPM [15] will be useful in formulating new hypotheses as it emphasizes that symptoms of PSU can be driven by different pathways triggered by distinct psychological processes. Additionally, new measurement scales should explore more specific behaviours and functions of smartphones. 

Bivariate correlations also showed that depression was positively associated with both PSU and average smartphone session length, whereby individuals with higher depression scores were more likely to exhibit PSU and use their smartphone for longer periods of time in one session. This supports the large body of research linking higher levels of depression to PSU [7,59]. These findings may be the result of individuals with higher depression levels using their smartphones to alleviate depressive symptoms such as negative mood and emotions. However, depression is a multifaceted condition and its relationship with PSU is likely to incorporate numerous factors—including the previously mentioned indirect effects such as sleep deprivation and work-related stress. Future research should aim to provide more detailed insights into these factors and their relationship with PSU, particularly around the work/family boundaries, as few studies have considered this aspect. In terms of PSU as a technological addiction, the study has made some constructive additions to the understanding of problematic use and its associated factors. Whilst the number of people with a genuine smartphone addiction is likely to be very small, it is important to understand the factors that can lead to addiction to develop appropriate interventions. The present study also served to examine the IPM [15], further examination of this model will allow for the development of a comprehensive theoretical framework of PSU. 

The study findings are very informative, but it is important to consider the study limitations. The use of a self-report method could inhibit the reliability of the study as participants may have underestimated their smartphone usage [75], particularly the number of short habitual checks [76]. Researchers [77,78] have recommended greater use of behavioural data to advance understanding of technological addictions. Corroborating behavioural data alongside self-report measures presents a key opportunity to move the field forward [77]. The internal consistency of the PSU measure was very good in this study and it has been used in previous research [28]; however, more validation is required to ensure that it can be utilised as a reliable measure of PSU. Additionally, there could also be issues with the construct being measured (i.e., is it PSU or attitudes towards smartphone use that is being measured?), there may also be overlapping of specific assessment constructs, for instance the BIS [72] and the MIGDS-SF [69] may measure the same core elements. Moving from research construct to formal disorder requires a stronger evidence base [24]. It has recently been argued that a general behavioural addiction category might be theoretically and clinically more defensible [24]. The present study adds to the debate concerning general behavioural addictions. In terms of sample size, the sample met the required parameters for multiple regression [79], however the sample size was relatively small in comparison to similar studies [16,28]. A larger sample size would further enable generalisation of the findings and potentially alleviate some of the ambiguous results, notably around excessive reassurance seeking. The study sample was mostly made up of young smartphone users so generalizability to older populations is problematic. Future studies should aim to recruit more diverse samples. 

Despite the limitations, the study was able to demonstrate the usefulness of the IPM [15] and presented numerous associations between PSU and personality traits. Age, impulsiveness, excessive reassurance seeking, and depression were associated with PSU. However, the present study and a small number of recent studies have raised questions over the relationship between extraversion and problematic use. It is important to continue development of a theoretical framework to underpin maladaptive behaviours. The study findings will benefit health practitioners and psychologists as they work towards developing treatments for the adverse effects of new technology. 

## Figures and Tables

**Table 1:** Summary of descriptive statistics.

Variable		Minimum	Maximum
Gender (Male, %)	42 (28.6)	-	-
Session Length (Minutes) (Mean, SD)	15.12 (12.02)	1	60
PSU (Mean, SD)	17.14 (5.69)	9	31
Impulsiveness (Mean, SD)	61.3 (9.38)	40	86
Excessive Reassurance (Mean, SD)	2.56 (1.48)	1	6.25
Extraversion (Mean, SD)	43.91 (11.84)	12	69
Depression (Mean, SD)	9.34 (6.63)	0	29

**Table 2:** Most frequently used smartphone features among participants.

Smartphone Feature	Frequency	Percentage
Messaging	58	39.5
Social Networking	56	38.1
Phone Calls	10	6.8
E-mail	7	4.8
Other	6	4.1
Gaming	5	3.4
Video Apps	2	1.4
Work	2	1.4
Shopping	1	0.7

**Table 3:** Correlations between the study variables.

	PSU (1)	Avg. SP Sess. Length (2)	Age (3)	Impulsiveness (4)	Extraversion (5)	Excessive Reassurance (6)	Depression (7)
1							
2	0.46 **						
3	−0.33 **	−0.44 **					
4	0.37 **	0.16	−0.13				
5	−0.10	0.00	−0.06	0.03			
6	0.36 **	0.22 **	−0.27 **	0.42 **	−0.13		
7	0.26 **	0.21 *	−0.12	0.36 **	−0.44 **	0.35 **	

** = *p* < 0.01, * = *p* < 0.05. Note: PSU = Problematic smartphone use, Avg. SP Sess. Length = Average smartphone session length, Excessive Reassurance = Excessive reassurance seeking.

**Table 4:** Bootstrapped ª multiple regression model of predictors of problematic smartphone use with BCa 95% confidence intervals (*N* = 147).

Variable	*B*	Standard Error	β	*t*	*p*	BCa 95% Confidence Intervals
Age	−0.11	0.03	−0.25	−3.30	0.001	−0.175–−0.046
Impulsiveness	0.16	0.05	0.26	3.02	0.003	0.066–0.252
Extraversion	−0.04	0.04	−0.08	−0.95	0.37	−0.123–0.041
Excessive Reassurance	0.59	0.31	0.16	1.82	0.057	−0.024–1.23
Depression	0.04	0.08	0.05	0.53	0.617	−0.129–0.207

Note: *R*^2^ = 0.25; Δ*R*^2^ = 0.23. ª Bootstrap results are based on 1000 bootstrap samples.
